# Managing Phenol Contents in Crop Plants by Phytochemical Farming and Breeding—Visions and Constraints

**DOI:** 10.3390/ijms11030807

**Published:** 2010-03-02

**Authors:** Dieter Treutter

**Affiliations:** Unit Fruit Science, Center of Life and Food Sciences Weihenstephan, Technische Universität München, Dürnast 2, D-85354 Freising, Germany; E-Mail: dieter.treutter@wzw.tum.de; Tel.: +49-8161-713-753

**Keywords:** flavonoids, phenylpropanoids, elicitor, stress, agricultural technology, apple, tomato, strawberry, lettuce, grapevine

## Abstract

Two main fields of interest form the background of actual demand for optimized levels of phenolic compounds in crop plants. These are human health and plant resistance to pathogens and to biotic and abiotic stress factors. A survey of agricultural technologies influencing the biosynthesis and accumulation of phenolic compounds in crop plants is presented, including observations on the effects of light, temperature, mineral nutrition, water management, grafting, elevated atmospheric CO_2_, growth and differentiation of the plant and application of elicitors, stimulating agents and plant activators. The underlying mechanisms are discussed with respect to carbohydrate availability, trade-offs to competing demands as well as to regulatory elements. Outlines are given for genetic engineering and plant breeding. Constraints and possible physiological feedbacks are considered for successful and sustainable application of agricultural techniques with respect to management of plant phenol profiles and concentrations.

## 1. Introduction

It was about 15 years ago that a booming interest in phenolic compounds, *i.e.*, phenylpropanoids and flavonoids, started among scientists from varying research fields. This was catalyzed by an increasing popularity of the potential benefits of that class of secondary plant metabolites for human health, and this ongoing phenomenon was triggered by some key publications [1,2] reflecting the change in appraisement of the physiology of these natural ingredients of plant foods. From a pharmaceutical point of view the effects of flavonoids on mammalian cells were extensively reviewed by Middleton *et al*. [3] summarizing the scientific activities at that time. They listed the interactions of flavonoids with mammalian enzyme systems, the modulatory role of flavonoids in inflammatory processes, the action of flavonoids as antiviral, antitoxic, cytoprotective and antioxidant compounds. Middleton *et al*. [3] also discussed the relation of flavonoids to coronary vascular diseases, to vitamin C and to cancer. The gene regulatory activity of flavonoid-rich plant extracts was highlighted by Gohil and Packer [4]. The scientific progress in relating these phenolic pharmaceuticals to nutrition may have been started much earlier with the work of Szent-Györgyi’s group who published the vitamin-like impact of pepper flavonoids [5]. Later on, all flavonoids with an effect on capillary permeability were subsumed as P-factors [6]. Pros and cons of these compounds in our diet were reviewed by Pierpoint [7] and Rogers [8], but are still a matter of debate [9–12]. The rising enthusiasm for these bioactive compounds, now also called nutraceuticals [13] is culminating in a huge number of scientific and even popular publications dealing with their health beneficial aspects. Fruits, vegetables and other crop plants seem to compete for a top ranking. Which may be the best: grape? cranberry? bilberry? apple? onion? tomato? pomegranate? green tea? soybean? buckwheat? carrot? artichoke? plum?…?

Based on the increasing knowledge of the biological activities of secondary plant constituents a number of commercial products have been introduced as food additives claiming health promoting properties and intending to counterbalance nutritional deficits of the Western diet as compared to Asian or Mediterranean food considered to be rich in phenolic compounds. Prominent examples are products containing grape seed meal or extracts. The respective bioactive components are catechins and proanthocyanidins [14], the latter are also called OPC (=oligomeric proanthocyanidins). In an example of commercial OPC-pellets high concentrations of monomeric catechins (10 mg/g) and soluble proanthocyanidins (15 mg/g) were estimated by HPLC-analysis (Treutter unpublished). Bread with grape seed additive contained despite of the heating 0.5 to 1.5 mg/g monomeric catechins and 0.2 to 0.5 mg/g proanthocyanidins (Treutter unpublished). Some food additives such as “Regulates” containing a wealth of phenolic compounds were analyzed more in detail and their antioxidant properties and immune modulating effects were demonstrated [15]. Within that context, it should be mentioned that there is an increasing interest in agricultural by-products which may be valuable as sources for bioactive compounds [16].

Some beverages are promoted as being of health promoting value because of their phenolic contents. A famous example is red wine with its phenolic constituents anthocyanins, proanthocyanidins and the stilbene resveratrol [17] considered to be responsible for the often cited “French paradox” which indicates a negative correlation between the wine consumed and the incidence of coronary heart disease [18]. The beneficial value of fruit juices is well accepted and attributed to various flavonoids (anthocyanins, flavonols, catechins and proanthocyanidins, phenylpropanoids). Intensively studied examples are apple juices [19–21], cranberry [22], black choke berry (*Aronia melanocarpa*) [23] pomegranate [24]. Efforts were made to increase the bioactive xanthohumol from hops in a German beer [25].

The profitable commercial use of phenolic compounds claiming their health benefits anticipate exact scientifically based knowledge about the daily intake being beneficial or essential or at least advisable for humans. This situation was critically reviewed by [26] who detailed facts and fiction with respect to nutraceuticals. Furthermore, despite consumers’ general acceptance of the health benefits of, for instance, flavonoids, the need to enhance the intake of phenolic compounds may be questioned. This is described as result of a study on consumer perceptions of flavonoids [27].

The contribution of some phenolic compounds to taste properties of plant food is known for the astringent mouth-feel due to condensed and hydrolysable tannins (proanthocyanidins, gallotannins, ellagitannis). Prominent examples of fruits are cider apples [28] and persimmon [28]. However, a certain amount of tannin-like components may be desirable for optimal taste of some beverages [30]. Apple juice producers in some regions of Germany use the juice of the proanthocyanidin rich service tree fruits (“Speierling”, *Sorbus domestica*) as a taste improving additive [31]. Recently, ethyl esters of hydroxybenzoic acids and hydroxycinnamic acids were identified as bitter compounds in wine [32]. Other phenolic compounds than tannins are considered responsible for the special organoleptic properties of olive oil, as well as its resistance to autoxidation [33].

Condensed tannins influence palatability and nutritive value of legumes [34] and protect ruminant animals against pasture bloat [35]. In a broad overview Mueller-Harvey [36] attempted to unravel the role of tannins in animal nutrition and examined the veterinary effects of these phenolic constituents of fodder plants. The legume sainfoin (*Onobrychis viciifolia*) is a good example for a pasture crop rich in diverse phenolic compounds [37] with beneficial properties for the health of ruminants. In particular, its anthelmintic properties were attributed to phenolic compounds [38]. The impact of tannins for human and animal health and nutrition was explained in detail [36,39].

Meanwhile the accepted wide range of beneficial effects of phenolic compounds initiated attempts to stimulate their accumulation in crop plants by agricultural technologies. Several reviews present and discuss the modification of phenolic profiles in plants for possible nutritional enhancement of our diet [40,41]. Schreiner and Huyskens-Keil [42] summarized the chances of targeted postharvest elicitor treatments to obtain fruits and vegetables enriched with health beneficial phytochemicals. The significance of anthocyanins in human diet and the occurrence in foods as well as selected agronomic influences were reviewed by de Pascual-Teresa and Sanchez-Ballesta [43]. An overview on agricultural practices for enhanced human health was given by Martínez-Ballesta *et al*. [44]. Ruiz-Rodriguez *et al*. [45] and Amarowicz *et al*. [46] summarized the influence of postharvest processing and storage on the content of phenolic compounds in foods.

Besides the nowadays desired enhancement of phenolic compounds in plant crops, in the past, several undesired phenolic compounds were removed by breeding and selection of varieties with a weak biosynthesis of secondary metabolites [47]. Critical in this regard are the furanocoumarins which act as phytoalexins and may induce contact dermatitis in sensitive people. The presence and levels of these compounds can be influenced by several plant treatments and storage conditions [48–50].

**Figure 1.**
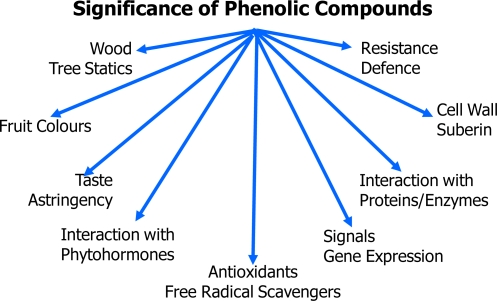
Multifunctionality of phenolic compounds.

Concurrently with the increasing popularity with respect to nutrition and human health the interest in phenolic compounds has also increased among plant scientists. Within the last four decades the opinion that phenolic metabolites are of major ecological and physiological importance has spread. The arguments against the old waste-product lobby were listed by [47] and [51]. Phenolic compounds contribute significantly to plant resistance against pests, pathogens and environmental stress [52]. They act as multifunctional metabolites in plants (Figure 1) being effective as sun screens, interacting with many enzymes and with growth regulators. They also may function as antioxidants in plants, a property which, however, is still a matter of debate [53]. Since information on insoluble phenolics bound to polymeric matrices is scarcely available, only data on the soluble pools being extractable with aqueous – organic solvents are considered here, taking into account that insoluble components may be released in the intestinal tract [54,55].

The aim of this article is to review visions and efforts being undertaken to manage the content and the profile of phenolic compounds in crop plants by cultivation technology, by using the genetic diversity of natural resources and by breeding. The outcome of those efforts is discussed with respect to their relevance on the basis of examples selected from literature. It is attempted to elucidate possible mechanisms behind the plant’s response to cultivation technologies. Further questions are treated: are there constraints, unexpected feed-back loops and physiological side effects? Which way shall we go in agricultural quality production?

## 2. Structures and Biosynthesis

**Figure 2.**
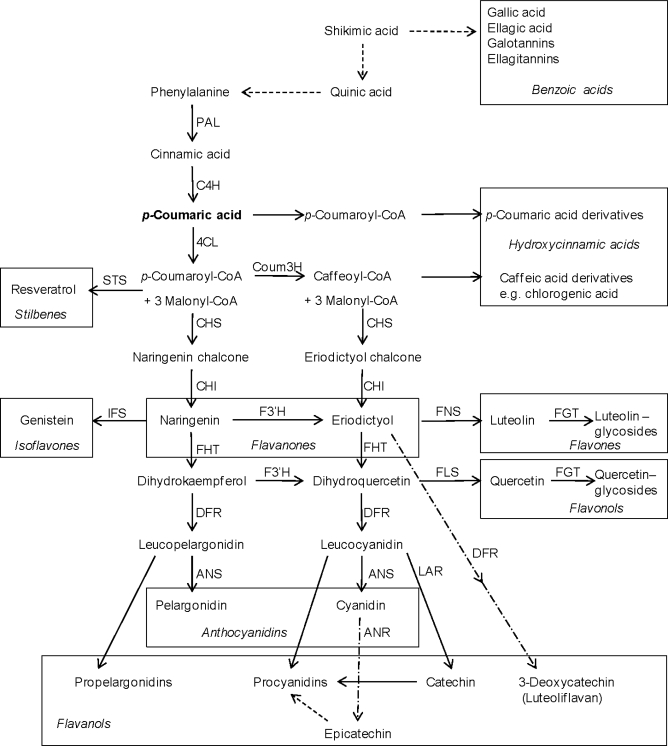
Simplified scheme of the biosynthesis of selected phenolic compounds. Abbreviations: ANR: anthocyanidin reductase, ANS: anthocyanidin synthase, C4H: cinnamate 4-hydroxylase, CHI: chalcone isomerase, CHS: chalcone synthase, 4CL: p-coumarate:CoA ligase, Coum3H: coumaroyl 3-hydroxylase, DFR: dihydroflavonol 4-reductase, F 3’-H: flavonoiod 3’-hydroxylase, FGT: flavonoid glycosyltransferase, FHT: flavanone 3-hydroxylase, FLS: flavonol synthase, FNS: flavone synthase, IFS: isoflavone synthase, LAR: leucoanthocyanidin reductase PAL, phenylalanine ammonia lyase.

Natural phenols in plants are derived from the shikimate pathway from which the gallic acid molecule and its polymeric gallotannins and ellagitannins are directly derived. As a further product from that pathway the amino acid phenylalanine is released. This amino acid is the precursor of phenylpropanoids and further phenolic classes derived from them. These are flavonoids, isoflavones, pterocarpans, stilbenes, coumarins, phenolamines, aurones, chalcones, lignans and lignin. A simplified example of flavonoid biosynthesis is given in Figure 2. For further details on biosynthesis and structures the reader is referred to [56–61].

## 3. Environmental, Nutritional, Agronomic and Developmental Clues Affecting Phenol Content in Crop Plants

### 3.1. Light Effects

It is well known that the secondary metabolism of plants is markedly influenced by changing environmental conditions. On the other hand phenolic profiles can be used for cultivar identification, which was applied for roses [62], geranium [63], gerbera [64], azalea [65], sweet cherries [66,67,68], sour cherries [69], pelargonium [70] and plums [71]. The phenolic profiles of plants of a given variety propagated under different environments can be adapted when transplanted to the same location and grown there for a few weeks. Thereafter, the new sprouts and the newly formed leaves of the same variety formerly propagated at different locations showed identical phenolic profiles [70] and cultivars could be differentiated. A study on grape varieties in Greece [72] could differentiate wines based on cultivar and geographical origin with application of chemometrics of their principal polyphenolic constituents. However, [73] stated that winemaking style and technology seem to be more decisive for the polyphenolic composition of red wines than other factors.

**Figure 3.**
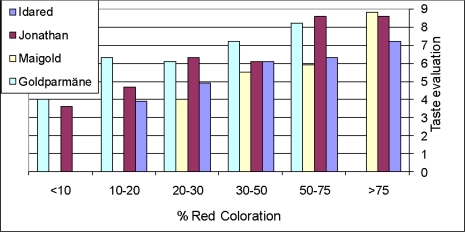
Relationship between red coloration of apple skin and taste evaluation (data from [74]).

The examples described above show the interest in stable phenolic fingerprints for variety discrimination. On the other hand, plant growers take advantage of environmental effects on crop yield and quality and, moreover, agricultural technologies have been developed to optimize Nature’s control. A prominent example is the management of the skin color of apple fruits which is equivalent of controlling anthocyanin biosynthesis and accumulation. The red skin of apple fruits is due to accumulation of anthocyanins and for weakly colored cultivars it is a suitable marker for both fruit development and inner fruit quality. Only those fruits developing on young, vigorous spurs and being exposed to sunlight exhibit strong sink potential for attraction of substantial amounts of assimilates from the leaves. A high sugar content of the fruits is an important parameter of fruit quality and is a prerequisite for the formation of flavor as well. Many apple cultivars produce anthocyanins as red pigments when exposed to sun light thus the red skin correlates with fruit quality as expected by the consumer (Figure 3)

Improved pigmentation of ‘Fuji’ apples was obtained by covering the orchard floor with light reflecting films [75]. The red pigmented fruit area increased from 11 to 38% corresponding to an increase in anthocyanin concentrations in the peel from 0.18 to 0.37 mg/g fresh weight. As a key enzyme for the accumulation of anthocyanins the activity of UDPGalactose:flavonoid-3-*O*-glucosyltransferase (UFGalT) was measured. The improved light conditions enhanced the enzyme activity from about 7 to 10 pkatal/mg protein. Other flavonoids were not influenced in that study [75]. The position of apple fruits on the tree is a main factor of light perception. Besides the accumulation of anthocyanins, the concentration of flavonols in apple skin also depends on the position in the tree [76] and reflects the percentage of red coloration and the sun exposure of the fruits. In contrast to the behavior of these flavonoids the concentrations of the dihydrochalcone phloridzin, of the catechins and of chlorogenic acid remained unaffected (Table 1). The accumulation of flavonols in apple skin when exposed to sunlight has been confirmed [77,78].

Beside a strong increase in anthocyanins and quercetin-glycosides as a result of sun-exposure, Hagen *et al*. [79] also found an about doubling of the values for epicatechin, procyanidins, phloridzin and an enhancement of chlorogenic acid in the peel of the apple cultivar ‘Aroma’. A postharvest irradiation treatment furthermore increased the total flavonoid concentrations but the individual compounds responded differently [79]. The impact of light exposure on the phenolic content and composition is reported in several other plants. Outer and inner leaves of lettuce heads differ in their concentrations of flavonoids [80]. Whereas outer leaves of the cultivars ‘Newton’ and ‘Rosalie’ exhibited concentrations of quercetin of 1.2 and 0.9 mg/g dry weight and luteolin of 0.11 and 0.16 mg/g dry weight, their inner counterparts accumulated only 3 to 7% of those values. When the inner leaves were opened and illuminated, an increase of the flavonoid levels up to the concentrations of the outer leaves took place. This coincides with an increase of chlorophyll and, probably, photosynthetic activity [80]. A short high light exposure (800 μmol/m^2^ s) for 1 day of young lettuce plants and analysis 3 days later revealed a pronounced increase of chlorogenic acid, chicoric acid, quercetin 3-*O*-glucoside and luteolin 7-*O*-glucoside [81].

The level of condensed tannins measured as tannin containing cells increased by increasing light intensity in leaves of *Lotus corniculatus* [82]. Soybean sprouts which are usually grown under dark conditions produce fewer amounts of isoflavones (0.56 mg/g fresh weight) as compared to green sprouts grown under light (1.38 mg/g fresh weight) [83]. Pruning and training of fruit trees and grape vines are commonly used practices to improve light perception of fruits and to increase red pigmentation. Fruit cluster thinning in grape skins cv. Syrah (*Vitis vinifera*) reduced the yield from 8 to 4 tons per hectare but nearly doubled the final concentration of the anthocyanin malvidin 3-glucoside in the fruits while flavonols remained unaffected [84]. Strawberry fruits do not seem to respond in such a strong way to light or shading [85]. No significant light effects on anthocyanin formation were found in ripening red raspberry fruits [86].

The effects of light on the accumulation of phenolic compounds in plant tissues may not only be explained by providing energy for carbon assimilation thus providing carbon resources for biosynthesis. It is furthermore the quality, namely the UV fractions, which stimulate the formation and accumulation of certain phenolic compounds in plants. Light and particularly UV-light enhances anthocyanin biosynthesis in apple fruit skins [87,88]. A further example is the cultivation of lettuce cv. ‘Lollo Rosso’ under plastic films with ultraviolet transparency [89]. When the concentration of anthocyanins in the leaves (μg/g fresh weight) were compared to plants grown under plastic film which blocks UV radiation, thus resembling a glass house, levels of the red pigments were more than doubled (Table 2). However, UV light also causes growth reduction by one third of the fresh weight and number of leaves was diminished by about 10%.

In another experiment [90], the UV-filter effect of a polycarbonate green house was assumed as the main reason for low levels of hydroxycinnamic acids and flavonoids in outer leaves of lettuce cv. Audran. In Table 3 the concentrations of phenolic compounds of these leaves 16 days after planting are compared with those from leaves grown outdoor. The general environmental conditions differed as follows: In the greenhouse, maximum photon photosynthetic flow (PPF) was 800 mE m^−2^ s^−1^ with the temperature between 15 and 29 °C; in the open air the maximum PPF was of 1,100 mE m m^−2^ s^−1^ with temperature between 10 and 20 °C.

UVB irradiation of *Betula sp.* seedlings for two months increased the concentrations of quercetin glycosides in the leaves [91]. When grapevine plants grown in the greenhouse were placed outside and exposed to UV-B light, the berries’ response was an accumulation of flavonols but not of hydroxycinnamic acids which results in a lack of UV-B screen whereas UV-A shielding by flavonols was improved [92]. Petunia plants exposed to UV-B showed a stimulated biosynthesis of flavonols with an increased quercetin/kaempferol ratio [93].

### 3.2. Temperature

**Figure 4.**
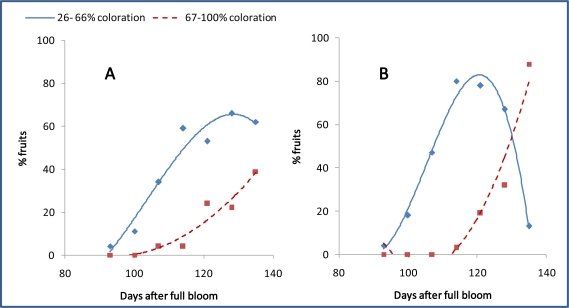
Development of red pigmentation of apple fruits cv. ‘Red Chief’ (% coloration) ripening under warm day (26 °C) and warm night (22 °C) conditions (A) and under warm day (26 °C) and cool night (11 °C) conditions (B). Data from [94].

Apples grown in warm climates often lack of sufficient skin coloration. This may mostly be due to high night temperatures during the last weeks prior to harvest. It was found that low night temperature during fruit ripening is a trigger for accumulation of red pigments in apple fruit skins [94] (Figure 4). Since consumers and retailers ask for red pigmented apple fruits the grower is enforced to postpone harvest under warm weather conditions. This may cause some disadvantages with respect to the storability of the fruits since other ripening related processes such as cell wall and starch degradation may have been accelerated before anthocyanin biosynthesis had started.

Low night temperatures also favour anthocyanin accumulation in grape berries [95]. At veraison plants were placed in a phytotron and grown under two day/night temperature regimes: 30 °C/30 °C and 30 °C/15 °C. Under the low night temperature conditions the berry skin accumulated up to 5.5 mg anthocyanins per g fresh weight whereas the concentration in the warm night variant was 4 mg/g fresh weight estimated at 45 days after veraison. Higher activity of PAL and in particular UFGT was found at low night temperatures coinciding with a high expression level of the *UFGT* gene [95]. In strawberries, high temperature during cultivation may generally promote the phenolic content of the fruits. This was shown for anthocyanins and *p*-coumaroyl glucose [96] (Table 4).

A short time stress treatment applying heat shock (40 °C for 10 min) or chilling (4 °C for 1 day) to young lettuce plants induced the accumulation of chlorogenic acid and chicoric acid while quercetin 3-*O*-glucoside and luteolin 7-*O*-glucoside only increased as a response to cold temperature [97]. In this context it has to be mentioned that red orange fruits (*Citrus sinensis*) accumulated anthocyanins in their juice vesicles during cold storage at 4 °C for a period of 75 days. This was measured as a linear increase starting from day 30 with a concentration of 1.5 mg/100 g fresh weight and reaching nearly 8 mg/100 g fresh weight at day 75 [98]. At the same time the transcripts of PAL, CHS, DFR and UFGT increased manifold. An accumulation of phenolic compounds was also found in apple during cold storage which was coupled with increasing PAL activity [99].

### 3.3. Mineral Nutrition

Maintenance of minerals is a prerequisite for providing co-factors for many enzymes of the phenylpropanoid and flavonoid pathway. Mg^2+^ and Mn^2+^ ions ensure the functioning of PAL, of CoA-ligases, and of methyltransferases [100–103]. Ca^2+^-deficiency induced anthocyanin accumulation in cabbage [104] and could promote lignification [105]. The metabolism of *Prunus* callus tissues *in vivo* was affected by Ca^2+^ deficiency and responded with an accumulation of phenolic stress metabolites [106]. Deficiency of phosphate in several plants leads to red coloration of the leaves which means accumulation of anthocyanins. In *Helianthus annuus* an accumulation of chlorogenic acid was observed [107] and in tomato fruits an additional enrichment of flavanones [108]. Boron deficient plants are also known to accumulate phenolic compounds which may be related to an activated pentose phosphate pathway in that situation [109]. In an *in vitro* system using grapevine callus, an increase in boron concentration in the nutrient medium from 0 to 600 μM was followed by a decline of catechins and proanthocyanidins by about 30% [110]. When AlCl_3_ was added to the medium in the same system of grapevine callus, an increase of the flavanols mentioned above by about 25% was measured. This may be attributed to a stress-type promotion as a response to aluminium [110].

The accumulation of phenolic compounds in plant tissues is often negatively affected by high N-nutrition. This was described for barley [111], for apricot fruits [112] and, recently, for tobacco [113]. It was also shown for red pine [114], and for *Pinus elliotti* [115]. N fertilisation reduced the concentration of individual phenolics in leaves of *Vaccinium myrtillus* [116]. Similarly, an increase of flavonols in tomatoes and *Arabidopsis thaliana* was confirmed by [117]. The fact of decreasing contents of phenolic compounds in leaves and needles of plants grown under high N supply was also described for several other species including *Abies grandis* [118], *Pinus sylvestris* [119], *Fagus sylvatica* [120], *Salix myrsinifolia* [121] *Betula pendula* [122] and *Betula pubescens* [123–126]. When grown at high level of N supply, leaves of potato plants contained significant lower amounts of chlorogenic acid and flavonols as compared to plants without additional N fertilization [127]. Among the factors influencing the red pigmentation of apple excessive nitrogen fertilization is commonly known as an inhibitory treatment. In an experiment on ‘Elstar Elshof’ apples [128] found a reduction of the main pigment cyanidin 3-galactoside by more than 40% after excessive N-fertilization (Table 5). Flavonols, catechins and phloridzin were affected showing a reduction by less than 20% whilst chlorogenic acid remained unchanged.

The effect of long-term N-supply on growth and phenolic compounds in the leaves of apple trees were examined in relationship to scab resistance [129]. The studies revealed a reduced accumulation of diverse phenol compounds except the hydroxycinnamic acids after fertilization with growth promoting amounts of nitrogen [129] (Table 6). The decrease of the phenolic concentrations by high N-fertilization was accompanied by a pronounced increase of susceptibility of the leaves to apple scab.

Whereas the phenolic levels in apple fruits and leaves suffer from excessive N-fertilization, strawberries seem to accumulate highest phenolic amounts when they were grown under conditions optimized for plant growth and fruit yield [130,131]. Such conditions comprise the use of a compost substrate and full strength fertilizer (NPK, 20/20/20) applied twice a week. All phenolic classes studied namely the anthocyanins, flavonols, hydroxycinamic acids and ellagic acid, responded in the same way (Table 7).

Conflicting results are presented by Anttonen *et al*. [132] who showed reduced phenolic concentrations in strawberry fruits by increasing nitrogen fertilization. However, from that experiment no information is available with respect to plant growth and yield. In *Solanum carolinense* an increase of phenolics was observed with increasing N-amendment [133]. Increased N nutrition of young birch seedlings reduced the level of soluble condensed tannins but increased its insoluble pool [91]. It is suggested that an increase in N, from deficiency to moderate, may enhance the accumulation of condensed tannins in the cell wall. A reduction of tannins in barley as a consequence of high N fertilization was reported by Sørensen *et al*. [134]. A more differentiated effect was found when wheat (*Triticum aestivum*) was extensively fertilized with nitrogen [135]: soluble phenolic acids increased in the straw but they were reduced in the corn. In the corn, additionally, a metabolic shift from p-coumaric acid towards ferulic acid occurred.

**Figure 5.**
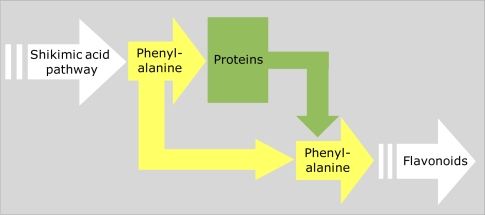
Different l-phenylalanine pools for the biosynthesis of phenolic compounds [141,142].

In cell cultures of grapevine and maple the quantity of anthocyanins was reduced when cultivated on high N containing medium [135,137]. In an experiment with apple skin discs anthocyanin formation coud be reduced by floating the tissue with urea while protein synthesis increased [138]. This was interpreted as an antagonistic relationship between anthocyanin development and protein synthesis. In leaves of *Hedera helix* turning red in the fall season both protein and anthocyanin biosynthesis occurred [139]. This is suggested to be possible because of the high sugar to protein ratio. Margna [140] provided evidence that phenylalanine may be the rate-limiting factor for phenyl-propanoid biosynthesis. However, protein degradation can be regarded as an alternative source for phenylalanine [141,142] (Figure 5). Recently, a downregulation of genes encoding for enzymes involved in the phenylpropanoid metabolism by nitrate as a proposed signal was described for *Arabidopsis* and tobacco [143,144].

Bongue-Bartelsman and Phillips [145] showed in case of nitrogen deficiency a modification of gene expression, which was not uniform. While the steady-state mRNA-levels of CHS and DFR in *Lycopersicon esculentum* increased under N deficiency stress, the steady-state levels of a CHI homologues band decreased at the same time. In the same plants also an accumulation of quercetin-3-*O*-glucoside and petunidin was found. A differentiated effect of nitrogen depletion on gene expression and product formation in different branches of the flavonoid pathway was described for *Arabidopsis* [146]. Decreasing activities of PAL were described in apple trees grown under high N and K supply [147]. Strissel *et al*. [148] confirmed this observation for high N supply in apple showing an impact on enzyme activity and on the content of metabolites.

### 3.4. Water Management and Irrigation

Plants grown under stress conditions often produce and accumulate phenolic stress metabolites [149–151]. Water deficiency is supposed to stimulate synthesis of phenolic stress metabolites in tomato. However, it was found that the concentration of flavonols on a fresh weight basis only reflected the reduced water content [152]. A similar effect was described for grape berries [153]. However, in years with water deficiency the flavonoid profile was shifted towards flavonols at the expense of flavanols [154]. A two years experiment with grape vine cv. ‘Tempranillo’ revealed that the highest amount of anthocyanins was produced under optimum water supply [155]. In a non-irrigated plot with pronounced yield reduction and reduced berry weight exceptionally only in one year a higher anthocyanin concentration was found when calculated on a fresh weight basis. When estimating the amount per berry, irrigation increased the accumulation of anthocyanins by more than 25% as compared to the non-irrigated variant. It may be concluded from these observations that with respect to water supply optimized cultivation technology favoring plant growth and fruit yield also assists the formation of anthocyanins. In a water logging experiment causing a growth retarding stress situation for cherry trees, [156] observed an increase of diverse phenolic compounds in the leaves.

### 3.5. Effect of Rootstocks

The propagation of many fruit tree varieties is only successful by grafting on compatible rootstocks. During centuries of fruit growing, rootstock genotypes have been selected which influence growth vigor of the combined tree consisting of two genetically different partners. Special scion/rootstock-combinations have been developed for optimized fertility, fruit yield and for high fruit quality which benefit from the rootstock’s influence on uptake and transport of water and minerals [157]. The interaction between the two partners also changes the hormonal situation in both the scion and the rootstock [158–160]. The effect of grafting on the phenolic compounds in the bark just above the union was studied for cherries and apricots with an accumulation of *p*-coumaroylglucose, of flavanones (*i.e.*, naringenin 7-glucoside), of the isoflavone genistin and of flavan 3-ols in cherries [161–163] and of flavan 3-ols in apricot [164]. The accumulation of those metabolites may indicate a lack of differentiation in the phloem and cambium region of the bark [165,166] since naringenin 7-glucoside and high concentrations of flavan 3-ols are potent markers of undifferentiated callus-like cells [167]. The altered phenolic profiles in the leaves of particular stressed *Prunus avium/P. cerasus* graft combinations which are characterized by an accumulation of chlorogenic acid, catechin and quercetin glycosides [168] may indicate an overall rootstock effect on the whole tree system. Recent studies on lemon trees revealed a pronounced accumulation of di-*C*-glucosyl diosmetin due to the use of sour orange rootstock instead of *Citrus macrophylla* [169]. The concentration increased from about 500 to more than 1,000 mg/L juice.

### 3.6. Elevated Atmospheric CO_2_

Global climatic change comes along with an increase of CO_2_ levels in the atmosphere. The increased supply of carbon may favor plant growth and provide resources for carbon based secondary metabolites. It was shown for tobacco that under limited nitrogen supply the ratio of secondary metabolites changed in favor of carbon-based phenolic compounds at the expense of alkaloids [113] when grown under elevated CO_2_ concentrations. Grapevine grown under elevated CO_2_ concentrations in a “Free-Air CO_2_–Enrichment” experiment produced more biomass and fruit yield increased. The general quality of the wine was not affected but the concentrations of anthocyanins and other flavonoids tended to increase [170]. High anthocyanin and phenolic content were also found in strawberry fruits [171] when plants were grown under CO_2_ enrichment conditions (ambient + 600 μmol/mol). Fruit *p*-coumaroylglucose increased from 417 to 735 μg/g dry weight, quercetin glycosides from 27 to 108 μg/g dry weight, kaempferol glycosides from 108 to 138 μg/g dry weight, cyanidin 3-glucoside from 561 to 1,153 μg/g dry weight, pelargonidin 3-glucoside from 2,124 to 3,669 μg/g dry weight. Elevated atmospheric CO_2_ under non-limiting nutrient and water supply increased condensed tannins and flavonol glycosides in birch seedlings [172] and total phenolics in *Pinus eliotti* [173]. The same trend was found in perennial grasses [174] and in *Plantago maritima* [175] as well as in several tropical trees [176]. In *Ligustrum vulgare* Tattini *et al*. [177] showed an accumulation of hydroxycinnamic acids and flavones as the CO_2_ assimilation increased. Elevated CO_2_ increased the concentration of the phenolic acids (125%), myricetin glycosides (118%), catechin derivatives (113%) and soluble condensed tannins (119%) by increasing their accumulation in the leaves of the silver birch trees, but decreased the flavone aglycones by growth dilution [178]. The altered flavonoid profiles under elevated CO_2_ may indicate that the physiological ageing of the respective trees was accelerated. Other experiments, however, failed to show an effect of elevated CO_2_ on phenolic compounds [179].

### 3.7. Differentiation and Development

**Figure 6.**
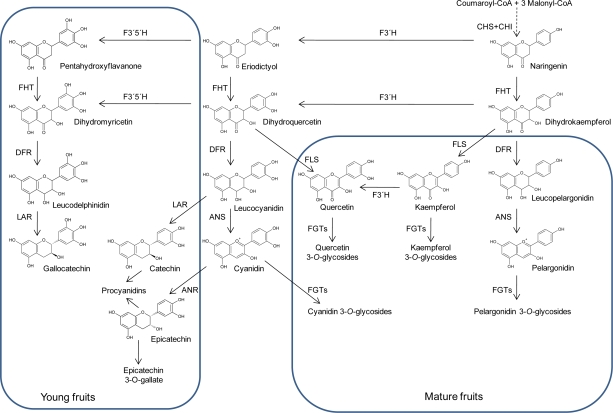
Flavonoid biosynthesis in strawberry fruits indicating development related pathways restricted to young and mature fruits, respectively [181]. Note: F3’5’H = favonoid 3’,5’-hydroxylase, FGTs = flavonoid glycosyl transferases, other abbreviations see Figure 2.

During tissue differentiation and organ development the phenolic profiles often undergo remarkable changes indicating that their metabolism is integrated into programs of growth and development. In the skin of young apple fruits cv ‘Golden Delicious’ at beginning of June the concentration of chlorogenic acid exhibits values of up to 30 μM/g dry weight dropping sharply down to 2 μM up to July and further reaching values near zero in the skin of mature fruits in September. Flavonol glycosides and phloridzin also show high concentrations in the skin of young fruits in June but with a plateau up to July and dropping down to about 30% of the initial values with concentrations in the mature fruits of 10 μM/g dry weight for phloridzin and 21 μM/g dry weight for flavonol glycosides [180]. Within the class of flavan 3-ols, catechin showed a similar behavior as flavonols but between 4 and 0.1 μM/g dry weight. Epicatechin and its oligomeric procyanidins started with about 10 and 7 μM/g dry weight, respectively, in June increasing to a maximum of about 20 and 26 μM/g dry weight, respectively, in July and dropping down to the initial values up to September. In developing strawberry fruits two distinct activity peaks could be demonstrated during fruit ripening at early and late developmental stages for most enzymes of the flavonoid pathway with the exception of flavonol synthase. The first activity peak corresponds to the formation of flavanols (catechin and procyanidins), while the second peak is clearly related to the accumulation of anthocyanins (pelargonidin glycosides) and flavonols (quercetin glycosides) [181] (Figure 6).

At the early stages of bilberry fruit development, procyanidins and quercetin were the major flavonoids, but the levels decreased dramatically during the progress of ripening. During the later stages of ripening, the content of anthocyanins strongly increased and they were the major flavonoids in the ripe berry [182]. Raspberry fruits also focus on anthocyanin formation during ripening [183]. Whereas ellagic acid showed high levels of nearly 300 μg/g fresh weight in unripe fruits its concentrations declined to 25% in fully ripe berries. Flavonols also decrease during ripening, for example quercetin 3-glucuronide from about 140 μg/g fresh weight to 25 μg/g fresh weight, while cyanidin-glycosides accumulate to more than 800 μg/g fresh weight with 60 and 83 μg/g fresh weight for cyanidin 3-sophoroside and cyanidin 3-rutinoside, respectively, and 380 μg/g fresh weight for cyanidin 3-glucoside and 320 μg/g fresh weight for cyanidin 3-glucosylrutinoside [183]. The inner bark of cherry shoots also showed developmental changes with respect to their flavonoid profiles [184]: In spring, the flavanone dihydrowogonin 7-glucoside reached concentrations of about 26 mg/g dry weight which fell down to 21 mg/g dry weight. During the same period, the isoflavone genistin increased from 1 to 4 mg/g dry weight.

When we attempt to actively influence phenolic profiles in plants we must keep in mind that biosynthesis and accumulation of phenolic compounds are mostly restricted to specialized cells and tissues and, moreover, at a subcellular level [150,185]. In close connection to their role as sun screens [186], the occurrence of flavonols in some plant organs is restricted to epidermal cells such as in grapevine leaves [187]. Infected cotton leaves were found to accumulate epidermal flavonoids (quercetin 3-*O*-glucoside, cyanidin 3-*O*-glucoside) in restricted areas where they are essential in protecting leaf tissue from light-dependent terpenoid phytoalexins [188].

**Figure 7.**
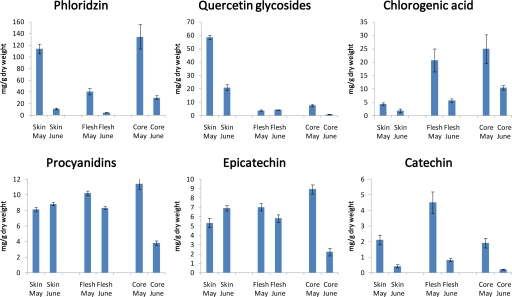
Tissue specific levels of phenolic compounds in young apple fruits cv. ‘Golden Delicious’ in May and June. Data from [180].

The skin of apple fruits being exposed to the environment may function as a protective layer thus expressing biosynthetic pathways different to those of the flesh and the core. In ‘Golden Delicious’ the glycosides of the flavonol quercetin are mainly accumulated in the skin whereas their concentrations in the inner parts of the fruit are quite low [180] (Figure 7). Chlorogenic acid on the other hand shows highest concentrations in the flesh and the core. Epicatechin and its oligomeric procyanidins are more or less equally distributed among the tissues but the level in the core decreases dramatically in June. The levels of phloridzin and catechin are seasonally regulated with highest values in the very young tissues in May.

### 3.8. Treatment of Plants with Elicitors, Stimulating Agents and Plant Activators

*Ethanol spray:* A perspective to improve grape berry color of anthocyanin deficient varieties or of grapes cultivated in inappropriate climate areas was offered by using ethanol as a spray [189]. Spraying a 5% ethanol solution on ‘Cabernet Sauvignon’ grapes at veraison (=time when berries turn from green to purple) increased anthocyanins up to 3-fold until harvest [189]. The improved accumulation was correlated with an induced expression of a glycosyltransferase gene UFGT (UDP glucose-flavonoid 3-*O*-glucosyltransferase). It is supposed that an unknown regulatory gene is active which is probably related to fruit ripening.

*Spraying of nutrients and plant activators*: Several reports describe the phenol enhancing effect of the nutrient solution ‘Brotomax’ which contains urea nitrogen, copper, manganese and zinc according to the manufacturer. After spraying it on olive trees [190], several phenolic compounds increased in the cortex of the stem: tyrosol from 4.2 mg/100 g fresh weight in the untreated cortex to 5.5 mg/100 g fresh weight in the treated one; catechin from 8.7 mg/100 g fresh weight to 9.6 mg/100 g fresh weight: oleuropein form 523 mg/100 g fresh weight to 651 mg/100 g fresh weight. Treating fruits of olive plants 50 days after anthesis had a beneficial effect on fruit size [191] and increased oleuropein depending on the variety by 50% to 300% reaching maximal values of 387 mg/100 g fresh weight. The phytoalexin scoparone (6,7-dimethoxycoumarin) accumulated in fruits of tangelo Nova, *Citrus aurantium*, and *Citrus paradisi* after treatment with Brotomax [192] enhancing their resistance to *Phytophthora parasitica*. By treatment of cucumber plants with the plant activator “Milsana”, the activity of chalcone synthase was induced resulting in a subcellular accumulation of a defense-related C-glycosyl flavonoid after infection by fungal pathogens [193–195]. Some plant activators may cause a wound-response at the surface which may include an accumulation of phenolic compounds. This was shown in a model experiment using surfactants [196].

*Cytokinin*: Several studies revealed promotion of phenylpropanoid bisoynthesis and accumulation in plant tissues by the action of cytokinins with an enhancement of PAL activity [197,198], an accumulation of total phenolics [199,200] and anthocyanins [201–204]. Cytokinin induced the accumulation of flavanones in *Prunus* callus cultures and phloem tissues of cherry shoots and alters the flavonoid profile of these tissues [166,206] (Table 8). An about 20% increase of the antimicrobial polymethoxyflavones (nobiletin, sinensetin, heptamethoxyflavone, tangeretin) was induced in the outermost tissue (flavedo and exocarp) of tangelo Nova fruits by treating with 6-benzylaminopurine. This application also enhanced the resistance of the fruit to *Phytophthora citrophthora* by 60%.

*Gibberellin GA_3_*: Treatment of grapevine *Vitis vinifera* flowers with a GA_3_ solution (20 ppm) influenced the phenolic profiles of the wine [207]. The response was not uniform among the varieties tested. The Hungarian cv ‘Kadarka’ showed strongest response with an increase of phenylpropanoids and flavonols by 52% and of anthocyanins by 229% as compared to wine from the non-treated control. This effect may be indirectly attributed to the altered growth and fruit set thus preventing infection by Botrytis [207]. More direct effects of gibberellins were recognized in tissue culture systems, where a decreasing effect of this hormone was observed [203,208–210]. Treatment of excised lettuce hypocotyls with GA_3_ suppressed PAL activity [211].

*Ethene*: Ethene has been known for a long time as an inducer of phenylpropanoid biosynthesis which may be related to a general wound and/or stress response. An ethene related increase of PAL activity was described for instance by [212–215] and an ethene induced accumulation of anthocyanins was described by [216–218]. The biosynthesis of anthocyanins and chlorogenic acid in ripening apple fruits may depend to some extent on the action of ethene since treatment of ‘Fuji’ apples with the ethene releasing agent ethephon enhanced activity of PAL and of chalcone isomerase as well as red peel color [219]. This treatment also stimulated ACC-oxidase activity and ethene formation indicating an effect on fruit ripening. When ethene receptors were blocked by 1-MCP (1-methylcyclopropene) the accumulation of phenolic compounds in apple fruits was impeded [220]. This observation is contradictory to the findings of Arakawa *et al*. [221] who proposed an ethene independent UV-B light inducible anthocyanin biosynthesis in apple fruits. The colouration of sweet cherries and the biosynthesis of chlorogenic acid in these fruits seem to be independent from ethene regulation [222].

*Methyl-Jasmonate:* Wang *et al*. [223] treated ripening raspberry fruits with methyl jasmonate which resulted in an increase of soluble sugars and a decrease in organic acids. The fruits of variety ‘Autumn Bliss’ also showed significant changes in flavonoid and ellagic acid contents. Flavonols (glycosides of quercetin and kaempferol) increased from 9.6 to 12.2 μg/g fresh weight, anthocyanins (glycosides of cyanidin) from 550 to 700 μg/g fresh weight and ellagic acid from 28 to 39 μg/g fresh weight. Schreiner and Huyskens-Keil [42] listed stimulating effects of postharvest treatments with methyl jasmonate on the flavonoid formation in apple, potatoes, guava, mango, banana and papaya.

*Benzothiadiazole:* The plant activator, benzo-(1,2,3)-thiadiazole-7-carbothioic acid *S*-methyl ester (BTH), when sprayed on grape berries cv. Merlot at the end of veraison, led to a more than doubled anthocyanin content in the grape berry skin extract at harvest [224].

**Figure 8.**
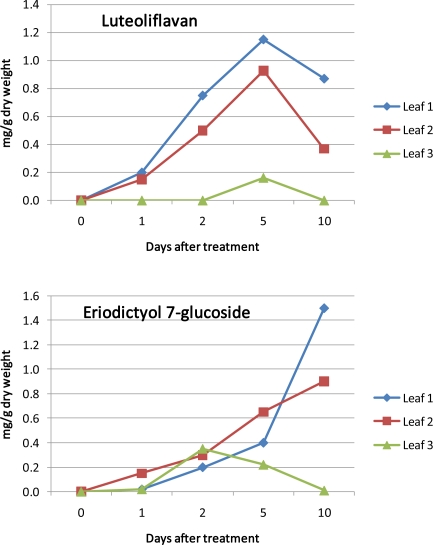
Transient alteration of flavonoid biosynthesis in young apple leaves treated with the bioregulator prohexadione-Ca. The newly formed 3-deoxycatechin luteoliflavan and the constitutive intermediary metabolite eriodictyol 7-glucoside accumulate most in the very young leaf no. 1 whereas the subsequent, older leaves at the shoot show weaker response. Data redrawn from [232].

*Bioregulator Prohexadione-Ca*: Among those agents activating plant defence the bioregulator prohexadione-Ca is a unique case since it is probably not acting via signalling pathways. Prohexadione-Ca reduced fruit set and berry weight of grapevine [225] and retards shoot growth of apples and pears by inhibition of the biosynthesis of hormonally active gibberellin [226]. Prohexadione-Ca is acting as a structural mimic of 2-oxoglutarate, and according to this property, it is able to inhibit dioxygenase enzymes, which require 2-oxoglutarate as a co-substrate [226]. Such enzymes are not only involved in formation of gibberellins but also in flavonoid biosynthesis [61]; and hence, prohexadione-Ca is able to alter flavonoid metabolism (Figure 6). As a consequence of this inhibition, several plants are able to re-direct accumulating metabolites towards unusual deoxyflavonoids [227–232] which show phytoalexin-like properties against several plant pathogens [233]. Novel flavonoids are formed in young leaves of apple [227,228] and pear [234] as well as in grapevine leaves and berries [230,235]. Even though for practical application in grape production the influence of prohexadione-Ca on the hormonal balance of the plant must be considered, which may lead, for instance, to reduced berry size and cluster compactness, the bioregulator can be applied as a tool to change the flavonoid composition of grapevine tissues. The mechanism of prohexadione-Ca action is not restricted to a redirection of the natural biosynthetic pathway leading to the back pressure at the level of flavanones and to the transient activation of an alternative pathway towards the formation of deoxycatechin (Figure 8). This disturbance of the metabolic balance is further characterized by an increase of transcripts of the key enzymes PAL and CHS [232] (Figure 2).

## 4. Mechanisms

### 4.1. Interacting Metabolic Pathways and Trade-offs

The mechanisms involved in the targeted accumulation of phenolic compounds in plants are complex and depend on the biosynthetic pathway to be controlled, on its localization and compartmentalization. Furthermore, diverse signal chains may be involved and the efficiency of measures depends on the accessibility of the respective target. Each primary signal such as abiotic or biotic elicitors will be followed by secondary messengers and an activated signal chain [236] inducing expression of genes, transcription to enzymes and formation and storage of metabolites. A prerequisite for accumulation of high amounts of phenolic compounds is the availability of resources and metabolic precursors from the primary metabolism.

Carbohydrate availability is a prerequisite for phenylpropanoid accumulation. When apple skin pieces were floated with sucrose, anthocyanins increased [237]. The accumulation of chlorogenic acid, catechin and quercetin 3-glucoside in stressed cherry leaves was associated with an accumulation of starch representing a potent carbohydrate pool [168]. Prunus callus cultures produced increasing amounts of flavanones and flavanols with increasing supply of sucrose in the growing medium [166]. *In vivo* apple shoot tip cultures also showed a pronounced accumulation of flavonoids. In that case it was flavanols and quercetin-glycosides [238]. Carter *et al*. [239] found in experiments on *Lotus corniculatus* that an elevated atmospheric level of CO_2_ increased the concentrations of nonstructural carbohydrates in the leaves correlating with higher concentrations of condensed tannins. Thus, trade-offs between plant growth and secondary metabolism may counteract with the management of phenolic compounds for agricultural purpose. Underlying hypotheses were recently commented by Matyssek *et al*. [240].

The accumulation of phenolic compounds in plant tissues under stress conditions may be explained by the action of elicitors stimulating signal chains towards biosynthesis and accumulation of metabolites. Another mechanism behind a stress related enhancement of phenylpropanoid biosynthesis may be driven by a deficiency in reduced pyridine nucleotides (NADPH). In that case, it is supposed that the pentose phosphate pathway is stimulated for providing NADPH thus releasing substrates for shikimate and phenylpropanoid biosynthesis. In model experiments using methylene blue for reconverting the reduced nucleotide to its oxidized form, anthocyanin formation was increased in apple [237] and flavanone accumulation took place in *Prunus* callus [167]. However, an inhibition of the citrate cycle was not effective for channeling hexose molecules via the pentose phosphate pathway towards the anthocyanin biosynthesis in apple [241].

An interesting link between primary and secondary metabolism was recently discussed by Lattanzio *et al*. [242] which couples the accumulation of the stress metabolite proline with the energy transfer towards phenylpropanoid biosynthesis via the oxidative pentose phosphate pathway [243]. Under several conditions of stress (pathogen infection, nutrient deficiency), the plant is forced to accumulate a large quantity of free proline. Its synthesis is accompanied by the oxidation of NADPH. An increased NADP^+^/NADPH ratio is likely to enhance activity of the oxidative pentose phosphate pathway providing precursors for phenolic biosynthesis via the shikimic acid pathway. The alternating oxidation of NADPH by proline synthesis and reduction of NADP^+^ by the two oxidative steps of the oxidative pentose phosphate pathway serve to link both pathways and thereby facilitate the continuation of high rates of proline synthesis during stress and lead to a simultaneous accumulation of phenolic compounds. Furthermore, it was shown that an application of proline to the nutrient medium of *in vivo* grown oregano plants elicited the accumulation of rosmarinic acid and other phenolic compounds in that plant [244]. It is suggested that mitochondrial proline oxidation could drive the oxidative pentose phosphate pathway by recycling glutamic acid into the cytosol to generate a proline redox cycle.

### 4.2. Regulatory Elements

The regulation of the biosynthesis of phenolic compounds is complex and in part undertaken by spatial organization and subcellular channeling. The consecutive enzymes of phenylpropanoid and flavonoid biosynthesis are supposed to be organized into linear or globular multienzyme complexes that can be associated with membranes or form metabolons [57,245,246]. Subcellular transport and channeling of metabolites functions with the aid of transporters and vesicles which are co-regulated with structural genes [247,248]. The enzymes are often present as multiple isoforms with varying subcellular or tissue-specific localization or different substrate specificities [245]. The corresponding structural genes are arranged as multigene families [251]. Single members of these families are differently regulated by transcription factors as well as by environmental clues [82]. Additionally some members are also differently expressed in different organs and tissues [249].

Transcriptional regulation is the major controlling step for secondary metabolite pathways [248,252–254]. The transcription factors involved in regulating anthocyanin and proanthocyanidin biosynthesis of *Arabidopsis* were identified as interacting MYB and bHLH type activators or repressors of the biosynthetic genes. Additionally, WD40 proteins are assisting the process. Different sets of interacting transcription factors were suggested to regulate genes encoding early or late biosynthetic steps, respectively [255,256]. The accumulation of phenolic compounds may be linked with the control of several other morphogenic and physiological processes [248]. The WD40 and HLH regulators seem to be involved in multiple processes whereas different MYB proteins act more specifically to regulate specific pathways. Transcription factors may therefore play a key role in coordinating secondary metabolism with plant differentiation and development [257,258]. For instance, a MYB factor found to be active in strawberry fruits may repress transcription in order to balance the levels of anthocyanin pigments produced at the late stages of strawberry fruit maturation [258]. A regulatory network of negative and positive feedback of gene expression might control and fine-tune the biosynthesis of anthocyanin and proanthocyanidin in *Arabidopsis* [259]. MYB type transcription factors were also shown to mediate environmental impacts such as mineral nutrient depletion as well as high light intensity on anthocyanin formation [146].

Some knowledge could also be gained on the regulation of anthocyanin accumulation in apples. Evidences that anthocyanin biosynthesis in apple skin depend on mRNA formation were discussed some times ago by Faust [138] who inhibited anthocyanin formation by several antibiotics. Meanwhile it is well understood that during apple fruit ripening and coloration, most of the structural genes of anthocyanin biosynthesis are induced and contribute to increase of red pigments [260–262]. It is further known that UV-B irradiation of the fruits and low temperatures increased the enzymatic activities of phenylalanine ammonia lyase and chalcone isomerase as well as the transcript levels of CHS, FHT, DFR, ANS, and UFGT [263–265]. Anthocyanin concentrations were found to positively correlate with UFGalT activities in fruit peel [75]. Environmental effects on the red pigmentation of apple fruits can be attributed to the function of transcription factors. A transcription factor MdMYBA was identified which controls the anthocyanin biosynthesis and is inducible by UV-light and cold temperatures [265]. Its expression is specifically regulated depending on the tissue and the variety with a higher expression in a deep-red variety as compared to a pale-red one. Based on these results on the MYB transcription factors in apple, there could be at least two loci of anthocyanin-related MYB transcription factors in apple, one corresponding to the coloration of the cortex of redfleshed cultivars and the other to the skin color of white-fleshed cultivars. The anthocyanin promoting effect of cold night temperatures may be supported by a reduced loss of sugars through respiration, resulting in a flow of metabolic precursors towards the biosynthesis of anthocyanins [88]. This expression pattern in apple differs from that in grape (*Vitis vinifera* and *V. labruscana*), in which UFGT induction during anthocyanin accumulation is a key regulatory step in the development of red coloration [266]. In grape, two kinds of MYB transcription factors regulate phenylpropanoid biosynthesis and show different expression patterns during berry ripening [267,268].

## 5. Managing Phenol Contents by Plant Breeding and Selection

The creation of new varieties of a given crop plant is a powerful tool to enrich our food with beneficial secondary metabolites and to increase the plant’s resistance against pathogens and pests or for utilizing any other advantage of a phenol-rich plant. This goal can be achieved by classical breeding if natural resources of high diversity are available. In the last century, classical breeding methods were applied to create ornamental plants with a vast diversity of flower colors based on profiles of anthocyanins with a range of flavonoid co-pigments (e.g., [269]), of aurones and chalcones [56,270]. The driving force for research on flavonoids and in particular on anthocyanins may have been their brilliance as flower pigments in ornamentals. The work done on flower colors gave initial insight into the genetics and biosynthesis of flavonoids [271,272]. This formed the basis of our current knowledge on molecular biology and regulatory genes [273,274].

### 5.1. Genetic Engineering

Outlines of different approaches for directing the flavonoid biosynthesis in crop plants by genetic engineering were presented by [275–278]. The most prominent strategies are shortly explained using tomato as an example. In order to improve the content of phenolic compounds as additional nutraceuticals beside the carotenoids being the main beneficial ingredients of tomato fruit, several strategies were developed and at least partially realized. For getting high-flavonol tomatoes the following transgenic strategies were attempted:

- overexpression of petunia chalcone isomerase
- heterologous expression of the maize transcription factor genes *LC* and *C1*
- ○ fruit-specific RNAi-mediated (RNA interference) suppression of the regulator gene *DET1*

Novel pathways using metabolites of phenylpropanoid and flavonoid biosynthesis have to be introduced for getting tomato fruits containing isoflavones and stilbenes. This was realized by using the following strategies:

○ overexpression of a soybean isoflavone synthase gene
○ overexpression of a stilbene synthase gene

Overexpression of petunia chalcone isomerase in tomato results in 78 fold increased levels of flavonols, mainly rutin, in the skin [279].

Simultaneous expression of the two regulatory genes *LC* and *C1* was required and sufficient to up-regulate the flavonoid pathway in transgenic tomato fruit flesh [280]. The ripe fruits of the transgenic *LC/C1* tomatoes accumulated high levels of flavonols. After hydrolysis of the glycosides, the concentrations of the aglycone kaempferol rose from about 2 mg/kg fresh weight in the non-transgenic fruit up to 60 mg/kg fresh weight in the transgenic ones. The corresponding values for the flavanone naringenin were about 20 mg/kg fresh weight and 60 mg/kg fresh weight. The expression of the specific anthocyanin regulating snapdragon transcription factors, Del and Ros1, induced the accumulation of high levels of anthocyanins in tomato [281]. The reasons underlying the success of this regulatory combination are partly explained by high levels of gene induction and by activating a broad spectrum of genes. *DET1* (*DE-ETIOLATED1*) is another regulatory gene which represses several signaling pathways controlled by light. Mutations in this gene are responsible for higher pigmentation of tomatoes due to elevated levels of both flavonoids and carotenoids. Using fruit-specific promoters combined with RNA interference (RNAi) technology tomato plants could be generated with degradation of *DET1* transcripts in their fruits [282]. In these transgenic lines fruits were found with concentrations for chlorogenic acid, for quercetin glycosides and for naringenin-chalcone more than doubled as compared to the wild type control. Other quality parameters such as fruit size and soluble solids were largely unchanged. When tomato plants were transformed with a soybean isoflavone synthase (*GmIFS2*) under the control of the cauliflower mosaic virus 35S promoter, substantial amounts of genistin (up to 90 nmol/g FW) were found in leaves, while the levels were marginally detectable (less than 0.5 nmol/g FW) in fruit peels [283]. In potatoes a 4-fold increase of the anthocyanins derived from petunidin and pelargonidin could be achieved by overexpression of a DNA encoding dihydroflavonol 4-reductase (DFR) [284]. A stilbene synthase gene was successfully introduced in apple plants [285] and the fruits produced the stilbene resveratrol-glucoside piceid [286].

Commercially relevant are the transgenic ornamentals which are already introduced into the market. Since the first transgenic petunia with pelargonidin type flower color was generated [287] many efforts were achieved. Of special interest are blue colored flowers such as the blue carnation and the blue rose where the shift towards the blue color was realized by increasing the B-ring hydroxylation pattern and accumulation the delphinidin type anthocyanins as a result of flavonoid 3’,5’-hydroxylase gene overexpression [288]. A general enhancement of anthocyanin production in petunia could be achieved by altering competition for substrate between flavonol synthase and dihydroflavonol 4-reductase [289]. On the basis of pigment and co-pigment analyses of *Kalanchoë blossfeldiana* varieties the introduction of acyl transferases is proposed as an approach to flowers with bluish colors [290].

Metabolic engineering strategies rely on the current knowledge. Thus, if the knowledge on biosynthetic pathways is incomplete, a proposed strategy may fail. One exciting example is the attempt to create red forsythia flowers by introducing dihydroflavonol 4-reductase and anthocyanidin synthase genes [291] which results in at that time unexplainable accumulation of epicatechin instead of anthocyanins. Some months later, this observation became explained by the discovery of the ANR enzyme reducing cyanidin to epicatechin [292].

In an outline on molecular breeding for color, flavor and fragrance Gutterson [293] stated in 1993 that *“the applications of molecular breeding in the near term will probably be few, and they will be focused on those crops where the economic reward can justify the technical investment cost”*. This may remain true for a while since transformation techniques and regeneration of transformed tissues to complete plants still have to be developed.

### 5.2. Diversity in Existing Varieties as a Prerequisite for Breeding

**Figure 9.**
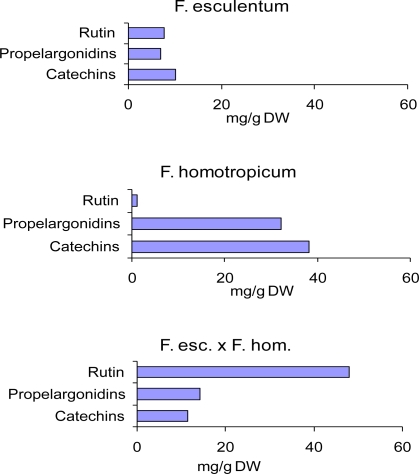
Phenolic compounds in buckwheat seeds (mg/g dry weight) from a breeding programme using *Fagopyrum esculentum* (F. esc.) and *F. homotropicum* (F. hom.) as parental plants with extraordinary contents of the flavonol rutin in interspecific hybrids. Data from [302].

In a trial with strawberries which were cultivated under several varying environmental conditions and cultivation methods like conventional and organical it could be shown that the variability of the phenolic contents among the varieties was much higher than the effect of cultivation techniques to be expected [132]. Variety and provenience dependent variations in phenol concentrations of apple were formerly summarized [294]. In a recent study on cider apples, pronounced concentration variability was found for most phenolic compounds [295]. The values in the pulp ranged from 48 to 227 mg/kg fresh weight for epicatechin, from 672 to 3,041 for procyanidins, from 61 to 724 for chlorogenic acid, 7 to 56 for phloridzin, 0.5 to 5 for quercitrin. The corresponding concentrations in the peel were: epicatechin, 11–81 mg/kg fresh weight; procyanidins, 360–929 mg/kg fresh; chlorogenic acid, 5–67 mg/kg fresh, phloridzin 7–123 mg/kg fresh; quercitrin, 3–29 mg/kg fresh. The flavonol content (quercetin glycosides) of commercial tomato fruits belonging to the species *Lycopersicon esculentum* reaches around 1.6 mg per fruit. A screening of wild species of the genus *Lycopersicon* revealed a widespread occurrence of key enzymes of the flavonoid biosynthesis [296] and *L. esculentum* x *L. penelli* accumulate up to 18 mg flavonols. Blueberry genotypes show a variation of the anthocyanin concentration ranging from 0.5 to 2.5 g/kg fresh weight [297]. Analyses of flavonols and hydroxycinnamic acids resulted in values between 0.2 and 1.2 g/kg fresh weight but with a negative correlation to fruit weight. The content of phenolic compounds varied widely between raspberry cultivars [85]. The quercetin content ranged from 0.32 to 1.55 mg/100 g fresh weight, ellagic acid from 38 to 118 mg/100 g fresh weight, anthocyanins from close to 0 (yellow cultivars) to 51 mg/100 g fresh weight. In a raspberry breeding programme the parent varieties *Rubus strigosus* ‘Latham’ and *R. idaeus ‘*Glen Moy’ exhibited 302 μg/mL and 206 μg/mL of cyanidin 3-sophoroside, respectively, as the main anthocyanin of the juice. In the progeny consisting of 188 plants, the concentrations of this pigment ranged from 39 μg/mL to 716 μg/mL. Compared to this broad variation, environmental effects on the anthocyanin level of raspberries were only marginal [298]. Anthocyanins in cranberry fruits from different genotypes ranged between 19.8 and 65.6 mg/100g fresh weight [299]. A screening of blackberry varieties [300] revealed concentrations of anthocyanins from 131 to 256 mg/100 g fresh weight, of procyanidins from 3.3 to 27 mg/100 g fresh weight, of ellagitannins from 7.7 to 27 mg/100 g fresh weight, of flavonols from 4 to 12 mg/100 g fresh weight. Spinach breeding lines show a broad range of special spinach-flavonoids from 0.8 to 2.2 mg/g fresh weight [301]. In a buckwheat (*Fagopyrum* sp.) breeding programme interspecific hybrids could be selected which exceed the parents in producing the bioactive flavonol rutin which is a strong quality factor of the buckwheat flour [302]. The powerful biosynthesis of catechins and propelargonidins characterising the species *Fagopyrum homotropicum* was redirected towards rutin formation in some hybrids with the common *F. esculentum* (Figure 9).

## 6. Constraints and Physiological Feedback

For optimized triggering the accumulation of target phenolic compounds in crop plants information is needed on the complete reaction chain starting with the primary elicitor and its effects on secondary signals followed by gene expression, transcription and activity of related enzymes and ending with the formation of metabolites. Possible side effects on other biosynthetic pathways must be taken into account. As described above (Section 3), a couple of tools are available and are practiced to a certain extent. However, a generally valid prediction of the plants’ response to a newly applied inductive method is scarcely possible and must take into account that the secondary metabolism of the respective plant is embedded into growth and differentiation. It may help to clarify first which role a given phenolic compound may play in the plant. One important function of flavonoids is the activity of a sunscreen [77,92,93] (and as photoprotective agents [303,304]). Many phenolic compounds act as resistance factors [52] with a multifunctional role [305] and are involved in adaptation to environmental stress conditions such as low or high temperatures [97,306]. These tasks imply the formation and accumulation in those plant tissues which are exposed to the environment and which have to be protected. Regarding red skinned apples, light exposure is leading to the desired result. UV irradiation of lettuce, however, may increase the phenolic levels in the outer leaves of a lettuce head which normally will not be eaten but thrown into the waste.

Depending on the mechanisms of induction or elicitation of the stimulation of biosynthesis it is furthermore essential to know if the respective constitutional pathway is active and if the constitutive enzymes are already working. This may be modified by development and differentiation of the plant or at least the respective organ since the phenylpropanoid and flavonoid pathways are integrated into these processes. For instance, UV-B induced flavonol accumulation in Petunia leaves declined with increasing leaf age [93]. With regards to a possible activation of the deoxyflavanol pathway by prohexadione-Ca it was found that only very young leaves exhibit a strong response (Figure 6). In older leaves the flavonoid pathway seems to be “silent” and cannot be activated by the bioregulator [232]. A similar temporary effect of the bioregulator treatment was also shown for grapevine berries and leaves [307]. The characteristic response of strawberry fruits to prohexadione-Ca treatment with the accumulation of the flavanone eriodictyol 7-glucoside and the 3-deoxycatechin luteoliflavan could only be induced at specific ontogenetic stages [235]. While flowers and mature green and red fruits only weakly responded to the treatment, the stage of small green fruit accumulated the novel compounds to a high level indicating a strong flavonoid biosynthesis at that developmental stage of the fruit.

**Figure 10.**
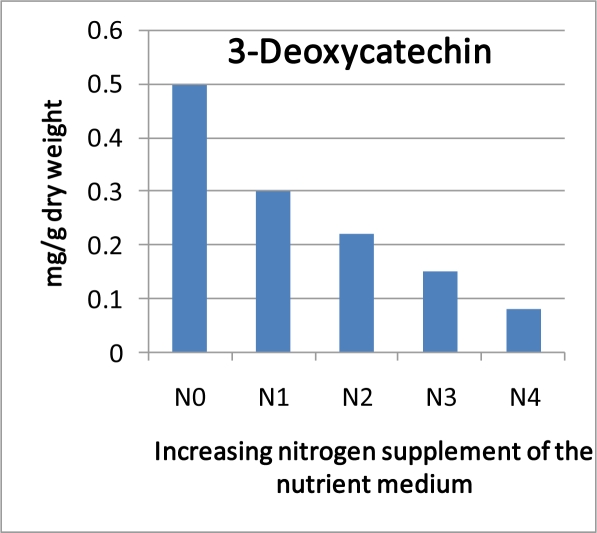
Effect of nitrogen nutrition on the prohexadione-Ca induced accumulation of 3-deoxycatechin (luteoliflavan). Redrawn from [309].

If the prerequisite of the available and active enzymes is fulfilled a further constraint could appear if resources are limited or trade-offs exist. A negative relationship between apple shoot growth and accumulation of phenolic compounds was reported formerly [308]. In *in-vitro* studies, this effect could be strengthened by high nitrogen supplement which dampens the prohexadione-Ca induced biosynthesis of 3-deoxycatechin in apple shoots [309] (Figure 10). In grapevine, only a moderate *N*-nutrition allowed a response to UV-light with regards to the accumulation of quercetin glycosides [310].

When the goal of an enrichment of phenolic compounds was reached it has to be anticipated that a physiological feedback may occur. The physiological activity of phenolic compounds with particular roles in growth related processes is debated since long [311,312]. Ferulic acid could act as an auxin antagonist during root formation of apple tree [313] and could affect hypocotyl growth of *Amaranthus* seedlings [314]. Naringenin 7-glucoside was isolated from peach buds and identified as a growth inhibiting factor possibly related to bud dormancy [315–317]. The differential effects of phenolic compounds on the activity of IAA-oxidases are well established with mono-phenols being promoting and catechol-type phenols being inhibiting [318–321]. The preservation of indoleacetic acid by catechin supplement in the nutrient solution of *Prunus* callus cultures may be responsible for pronounced changes in tissue differentiation and metabolism [322]. The effect of different cinnamic acid derivatives on indole acetic acid oxidation by peroxidase was intensively studied [323]. Interaction with plant hormones may explain the observed growth promotion of callus cultures by chlorogenic acid on auxin-free media [324,325]. It is obvious that flavonoids may regulate auxin transport in plants [326]. Beside that effect on the IAA-metabolism, phenolic compounds may also affect the basipolar transport of auxin. A stimulation of auxin movement was found for naringin [327]. An inhibition of oxidative phosporylation and of membrane bound ATP-formation was found for some flavonoids, such as naringenin [320,328,329]. Novel functions of flavonoids will be deduced from recent investigations on the accumulation of flavonoids in the nuclei of a wide range of species [185,330–332]. It is stated that nuclear flavonoids bound to histones are involved in epigenetically regulated modification of chromatin [333].

What may happen if phenolic compounds leave their vacuolar compartment? In incompatible graft unions of cherries, naringenin 7-glucoside was found to accumulate as a stress metabolite [334]. It could be shown that such an abnormal accumulation may affect cellular differentiation and secondary metabolism after leaching from its main compartments, the vacuole or cytoplasmic vesicles, in case of further stress related membrane dysfunction. When *Prunus avium* and *P. cerasus* cells were treated with naringenin 7-glucoside, vessel differentiation was inhibited and instead of this, highly vacuolated cells were formed with a strong biosynthesis of catechins and proanthocyanidins [167].

As long as phenolic compounds are put away in vacuoles or vesicles they may not exhibit significant effects on any physiological process of the respective plant tissue. However, in stress situations membrane integrity may be affected [106], thus, their phenolic content may be released and come in contact with the metabolic and regulatory machinery of the cell [335]. The cellular differentiation of *Prunus* callus cultures could be reorganized by application of naringenin 7-glucoside via the nutrient solution [106,167]. The response of the plant tissue on the invasion of an unusual or foreign phenolic compound depends on its chemical structure and physiological activity. Whereas naringenin 7-glucoside increased membrane permeability, reduced tissue growth, and caused a change in cellular differentiation [167,336], the flavan-3ol catechin was found to stabilize membranes and counteracted a growth inhibiting effect of abscisic acid [337]. Catechin was furthermore shown to promote growth of several plant tissues, including *Prunus* callus [322] and in pedicels of beech *Fagus sylvatica* [337]. From the viewpoint of a plant grower both positive and negative effects may appear. The possible antioxidant activity of phenolic compounds may be designated as a positive one. The detrimental effect of the free radical releasing paraquat molecule could be reduced by catechins in several plant models [338].

Morphological effects induced by the flavones isovitexin were also reported to occur in a non-glycosylating *Silene pratensis* genotype [102]. Furthermore, allelopathic plant-plant and plant-microbe interactions [339] may be expected. Phenolic compounds in root exudates of several plants are key signaling components in the symbiosis between different host plant species and their ecto- or endo-symbiotic fungal partners. Furthermore, upon interaction with their fungal symbiotic partners the metabolic profiles of the host plant roots can be changed. [340–350]. Therefore, cultivation techniques and environmental factors affecting the phenolic profiles of such plants may influence symbiosis and by that indirectly plant growth as well.

## 7. Conclusions

Several approaches for managing the contents and profiles of phenolic compounds in crop plants are presented. Among the cultivation technologies, the application of controlled mild stress conditions is sometimes propagated and several ideas are born from *in-vitro* experiments using tissue cultures or young seedlings but the up-scaling to a marketable produce often remains obscure. Results obtained by model experiments have to be thoroughly interpreted and carefully extrapolated to commonly used production methods.

**Figure 11.**
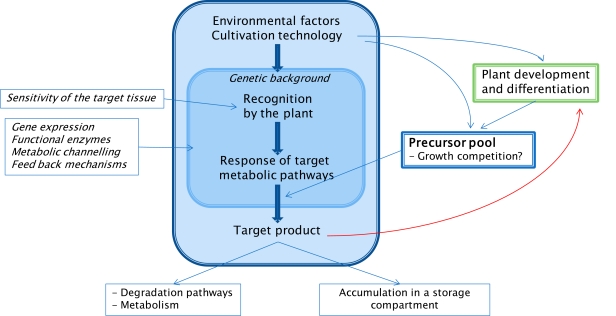
Route of targeted pytochemical farming indicating constraints and physiological feed back.

The main route of targeted phytochemical farming (Figure 11) starts from the environmental factors modified by agricultural technology which must be recognized by the plant requiring sensitivity of the respective tissue. This step must be followed by a metabolic response depending on the expression of related genes which has to be transcribed to functional enzymes. Metabolic channeling may then direct to the accumulation of the target product if not disturbed by feedback mechanisms or by further metabolism or degradation. The farmers’ activities and/or changing environments also control growth and differentiation of the plant, thereby modifying precursor pools of the targeted secondary metabolism. The requested bioactive compound may exhibit physiological activity, thus affecting plant development and ecological performance. Increased resistance to biotic and/or abiotic stress situations may crop up as an added value of accumulating phenolic compounds.

Before such efforts in adapting cultivation technologies can be advised to the plant grower we should know:

○ the beneficial effects of target chemicals
○ the beneficial concentrations
  • for plant resistance
  • for human or animal health
○ environmental impacts on biosynthesis and metabolism
○ the role of the target phytochemicals in the plant.

A further prerequisite is an expected financial return. If a particular phenolic composition and concentration could be achieved, the question is how sustainable is the commercial return? Consumers’ attitudes may change or a reappraisal of particular bioactive components may happen based on advances in scientific knowledge. A curious example is the loss of the value of apples’ red skin colour as a marker for inner quality. Red coloration of many fruits like apple and strawberries may portend high fruit quality and optimal ripening stage based on the often observed biosynthetic relation to developmental processes in such fruits. High taste quality correlates with red pigmentation of old weakly colored apple cultivars (Figure 3) [74]. This may account for the consumers’ preferences since about 50% of the consumers indicate fruit color as a main selection criterion [351–353]. This contributes to higher prices for apple fruits for instance grown in the mountains of South Tyrol with prices attained by the growers of 45 €-Cents per kg as compared to the price for fruits grown in the valley with 35 €-Cents per kg [354]. Another study in 1986 revealed a 10% higher price for ‘Roter Boskoop’ as compared to the ‘Gelber Boskoop’ and more than 60% higher price for red mutants of ‘Jonagold’ in comparison to the standard ‘Jonagold’ [355]. The higher economic value, however, enforced the fruit growers and breeders to focus on mutants which accumulate anthocyanins even under low light conditions. Since most of the other fruit characteristics remained unchanged the red skinned mutants replaced the original cultivars to a high extent. This was observed for the old apple varieties ‘Gelber Boskoop’, ‘Gravensteiner’, ‘James Grieve’ which have been replaced by red mutants. Among the new apple varieties ‘Jonagold’, ‘Elstar’, ‘Gala’, ‘Braeburn’, ‘Fuji’, ‘Topaz’, ‘Pinova’ many red mutants have been selected up to now [356–361]. By that kind of selection the value of the red apple skin as a useful marker for high fruit quality with optimal organoleptic properties is strongly reduced. This could be confirmed for red-skinned mutants of ‘Gala’ and ‘Jonagold’ where the correlation between red skin and ripening stage is poor [353]. Nevertheless, the red color of apples is paid for!

Despite such uncertainties with respect to the sustainability of a measure for phytochemical farming, it is inevitable to increase our knowledge on optimization and influencing the composition and enhancement of “bioactive” metabolites in plant foods (namely fruits and vegetables) by breeding (classical methods, genetic engineering), production technology, storage and processing. At the same time, the plant grower should keep an eye on the progress in nutritional science and nutrition medicine which may state the biologic activities of phenolic compounds more precisely. At the moment, it may not be recommended starting the production of plant foods with an attributed enhanced nutritional quality for a common market. However, the production of fresh “functional food” with defined health claims may be favorable for a premium market segment. In future, it may be expected for the global market that minimum quality of plant foods will be defined on the base of their content of bioactive components.

## Figures and Tables

**Table 1.** Red coloration of apple fruits and content of phenolic compounds (mg/g dry weight) in the skin as affected by fruit position in the tree. Data from [75].

**Position on tree**	**% Red coloration**	**Cyanidin - galactoside**	**Quercetin glycosides**	**Catechins**	**Phloridzin**	**Clorogenic acid**

Top	38.0	0.6	8.8	3.0	1.2	0.17
Outer west	20.5	0.3	6.8	3.5	1.2	0.21
Outer east	14.2	0.2	7.0	3.7	1.2	0.20
Inner	0.0	0.0	2.5	3.6	1.1	0.20

**Table 2.** Growth parameters and anthocyanin concentrations in green and red leaves of lettuce cv. ‘Lollo Rosso’ grown under plastic films with and without UV transparency. Data from [89].

**UV transparency of the polytunnel**	**Fresh weight of the lettuce**	**Leaf number**	**Plant sample**	**Anthocyanins (μg/g fresh weight)**

no	320	27	green leaves	25
no			red leaves	375
yes	190	23	green leaves	75
yes			red leaves	992

**Table 3.** Phenolic compounds (mg/g fresh weight) in outer leaves of lettuce grown in open air and in a polycarbonate greenhouse, 16 days after planting [90].

	**open air**	**greenhouse**

Chlorogenic acid	0.77	0.41
Chicoric acid	1.17	0.48
Quercetin glycosides	0.30	0.01

**Table 4.** Effect of plant growth temperature (day/night °C) on concentrations (μg/g) of phenolic compounds in fruit juice of strawberry cv. ‘Kent’; data from [96].

	**Temperature (day/night, °C)**			

Phenolic compound	18/22	25/12	25/22	30/22
Pelargonidin glycosides	449.1	623.1	880.5	1220.5
Cyanidin glycosides	36.5	42.4	45.3	65.6
*p*-Coumaroyl glucose	30.8	46.7	61.5	73.4
Quercetin glycosides	2.2	3.6	15.7	21.4
Kaempferol glycosides	2.4	3.4	4	6.2

**Table 5.** Effect of N-fertilization on N-content and on the content of phenolic compounds in apple skin (cv ‘Elstar’ mutant ‘Elshof’). Data from [128].

**Fertilization level**	**0**	**2**	**4**

N concentration in the fruits (mg/100g fresh weight)	32.0	41.1	54.1

Phenolic compounds in the skin (mg/g dry weight)			

Cyanidin 3-galactoside	1.1	0.86	0.64
Quercetin glycosides	4.9	4.8	4.3
Catechins	3.0	2.9	2.5
Phoridzin	0.86	0.95	0.75
Chlorogenic acid	0.047	0.053	0.045

**Table 6.** Phenolic compounds (mg/g dry weight) in young leaves of apple cv. ‘Golden Delicious’ as influenced by nitrogen fertilization with N1 as the conventional control and N3 as an excessive treatment. The C/N-ratios of the shoots indicate the different physiological situations. Data from [129].

	**N1**	**N3**

Shoots C/N ratio	55.0	43.0
Phloridzin (mg/g dw)	78.0	50.0
Flavonols (mg/g dw)	11.5	9.0
Phloretin (mg/g dw)	7.0	2.0
Hydroxycinnamic acids (mg/g dw)	1.5	1.5
Procyanidins (mg/g dw)	1.4	1.0
Catechins (mg/g dw)	0.6	0.3

**Table 7.** Phenolic compounds in fruit of strawberry cv ‘Honeoye’ as affected by fertilizer strength. Data from [131].

**Phenolic compounds**	**Fertilizer**		

μg/g fresh weight	none	half strength	full strength

Pelargonidin glycosides	807.0	855.1	923.5
Cyanidin glycosides	18.4	24.3	37.9
p-Coumaroyl glucose	47.0	49.3	70.5
Kaempferol glycosides	12.1	13.8	15.1
Ellagic acid	2.2	3.9	6.3

**Table 8.** Changes of flavonoid concentrations (% of untreated controls) in the inner bark (phloem) of *Prunus avium* shoots as a response to benzyladenine application. Data from [206].

***Prunus avium* cultivar**	**Naringenin 7-glucoside**	**Chrysin 7-glucoside**

Burlat	130	98
Sekunda	150	96
Kassins	180	70
Roße Schwarze Knorpelkirsche	200	70
Abels Späte	220	80
Büttners	250	75
Bigarreau von Ordingen	280	78
Delta	295	90
Königskirsche	300	92
Sam	320	88
Van	320	75
